# The *Drosophila* neurogenin Tap functionally interacts with the Wnt-PCP pathway to regulate neuronal extension and guidance

**DOI:** 10.1242/dev.134155

**Published:** 2016-08-01

**Authors:** Liqun Yuan, Shu Hu, Zeynep Okray, Xi Ren, Natalie De Geest, Annelies Claeys, Jiekun Yan, Eric Bellefroid, Bassem A. Hassan, Xiao-Jiang Quan

**Affiliations:** 1 VIB Center for the Biology of Disease, VIB, Leuven 3000, Belgium; 2 Center for Human Genetics, University of Leuven School of Medicine, Leuven 3000, Belgium; 3 Program in Molecular and Developmental Genetics, Doctoral School for Biomedical Sciences, University of Leuven School of Medicine, Leuven 3000, Belgium; 4 Medical College, Henan University of Science and Technology, Luoyang, Henan Province 471003, China; 5 Laboratoire de Génétique du Développement, Université Libre de Bruxelles, Institut de Biologie et de Médecine Moléculaires (IBMM), Gosselies 6041, Belgium

**Keywords:** Neurogenin, Axonal growth, Mushroom body, Wnt signaling

## Abstract

The neurogenin (Ngn) transcription factors control early neurogenesis and neurite outgrowth in mammalian cortex. In contrast to their proneural activity, their function in neurite growth is poorly understood. *Drosophila* has a single predicted Ngn homolog, Tap, of unknown function. Here we show that Tap is not a proneural protein in *Drosophila* but is required for proper axonal growth and guidance of neurons of the mushroom body, a neuropile required for associative learning and memory. Genetic and expression analyses suggest that Tap inhibits excessive axonal growth by fine regulation of the levels of the Wnt signaling adaptor protein Dishevelled.

## Abstract (precis)

**Summary:** Mammalian neurogenins are proneural factors, but the *Drosophila* homolog Tap is not, instead acting to prevent axonal outgrowth, likely by regulating the planar cell polarity pathway via Dishevelled.

## INTRODUCTION

Proper function of the nervous system is based on the production of a diversity of neuronal and glial cells, as well as the precise targeting of their axons and dendrites. A key transcription factor (TF) family in the control of neuronal cell fate commitment and neurite guidance is the neurogenin (Ngn) family. Ngn proteins belong to a structurally and functionally conserved basic helix-loop-helix (bHLH) superfamily. Ngns in vertebrates are sufficient to initiate neuronal cell fate in the central nervous system (CNS) (reviewed by Bertrand et al., 2002; Ma et al., 1998). By contrast, very little is known about the function of Ngn proteins in invertebrate systems. Gain-of-function analyses in *Drosophila* and vertebrate models suggest that during evolution a switch in proneural activity occurred between the Ngns and the highly related atonal family of bHLH TFs. Specifically, whereas Ngns are necessary and sufficient for the induction of neurogenesis in vertebrates, they cannot do so in flies. Conversely, in flies atonal-type proteins can induce neurogenesis, but fail to do so in vertebrates (Quan et al., 2004). A crucial test of this ‘evolutionary proneural switch hypothesis’ is whether *Drosophila* Ngns act as proneural genes in flies and vertebrates.

In addition to their proneural function, Ngns play various crucial roles in the development of the vertebrate nervous system, including regulating the outgrowth and targeting of both axons and dendrites (Hand et al., 2005; Hand and Polleux, 2011; reviewed by Yuan and Hassan, 2014). This regulation of neurite growth is independent of proneural activity, as the phosphorylation of a single tyrosine in mouse Ngn2 is necessary to specify dendritic morphology without interfering with cell fate commitment (Hand et al., 2005). However, the mechanism by which Ngn proteins regulate neurite guidance is poorly understood.

The *Drosophila* genome encodes a single Ngn family member, based on the conservation of family-defining residues in the bHLH domains (Hassan and Bellen, 2000), named Target of Pox neuro (Tap). Previous work suggests that Tap is expressed in secondary progenitors of both neurons and glia, called ganglion mother cells, in the CNS (Bush et al., 1996), as well as in putative support cells in the peripheral nervous system (PNS) during embryogenesis (Gautier et al., 1997). A previously reported putative *tap* mutant allele (Ledent et al., 1998) was later shown to be a mutation in a different gene called *blot* (Johnson et al., 1999). Therefore, the function of the *Drosophila* Ngn Tap remains uncharacterized.

Here, we generated a null mutant allele of *tap* by replacing the single coding exon of the *tap* gene with *Gal4*. Using expression, gain-of-function and loss-of-function analyses we show that whereas ectopic expression of Tap in vertebrates can induce neurogenesis, *tap* is not a proneural gene in flies, consistent with the evolutionary proneural switch hypothesis. Instead, Tap is required to prevent overgrowth of axons during brain development, at least in part through the activity of the axonal Wnt-planar cell polarity (PCP) pathway, by fine tuning the levels of the Wnt signaling adaptor protein Dishevelled (Dsh).

## RESULTS AND DISCUSSION

### Tap is the only Ngn homolog in *Drosophila*

**Fig. 1.**
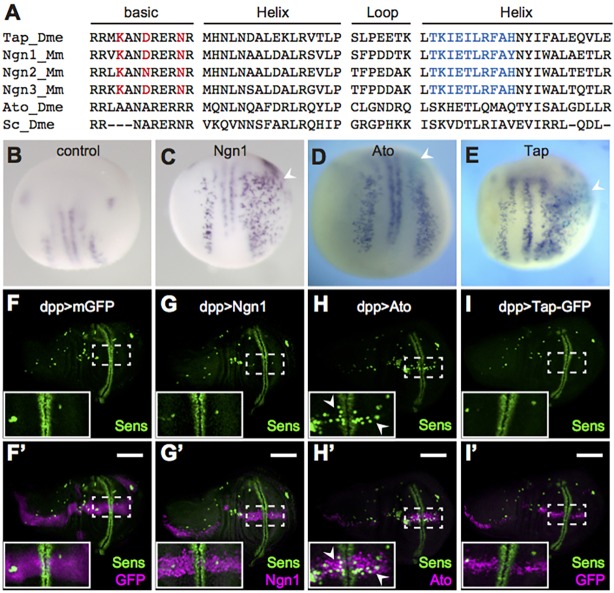
**Tap is the Ngn homolog in *Drosophila*.** (A) Sequence comparison among bHLH domains of fly Tap, mouse Ngn family, fly Atonal (Ato) and Scute (Sc). Three residues in the basic domain (red) were identified as defining the proneural function of Ngn and Ato in vertebrates and flies, respectively. The ten residues in the second helix domain (blue) are also class specific (Quan et al., 2004). Dme, *Drosophila melanogaster*; Mm, *Mus musculus*. (B-E) *In situ* hybridization of *N-tubulin* is used to visualize neurogenesis in *Xenopus* embryos upon either no injection (B) or injection of mRNA of mouse *Ngn1* (C) or *Drosophila ato* (D) or *tap* (E) (arrowheads). (F-I′) The distribution of neuronal precursors (Sens, green) in L3 wing discs. CD8::GFP (F,F′), mouse Ngn1 (G,G′), *Drosophila* Ato (H,H′) or Tap-GFP (I,I′) is ectopically expressed in the anterior-posterior axis of wing discs (magenta), driven by *dpp-Gal4*. Boxed regions are magnified in insets, where arrowheads indicate ectopic sensory organ precursors. Scale bars: 100 μm.

*Drosophila* Tap shares significant identity (∼70%) in the bHLH domain with mouse and human Ngns (Fig. 1A), as compared with *Drosophila* Atonal or Scute. To test if Tap, like its vertebrate counterparts, has proneural activity, we injected *tap* mRNA into one cell of two-cell stage *Xenopus* embryos and assessed neurogenesis by staining for neuronal markers. Like mouse Ngn1, but unlike fly Atonal, Tap efficiently induces neurogenesis in this system (Fig. 1B-E). Conversely, when ectopically expressed in a classic *Drosophila* proneural assay Tap – like Ngn1, and in contrast to Atonal – fails to induce neurogenesis (Fig. 1F-I). These data suggest that Tap may not be a proneural protein in flies, even though it has neural induction potential in vertebrates as expected for a bona fide Ngn protein.

### Tap is widely expressed in the nervous system during development

**Fig. 2.**
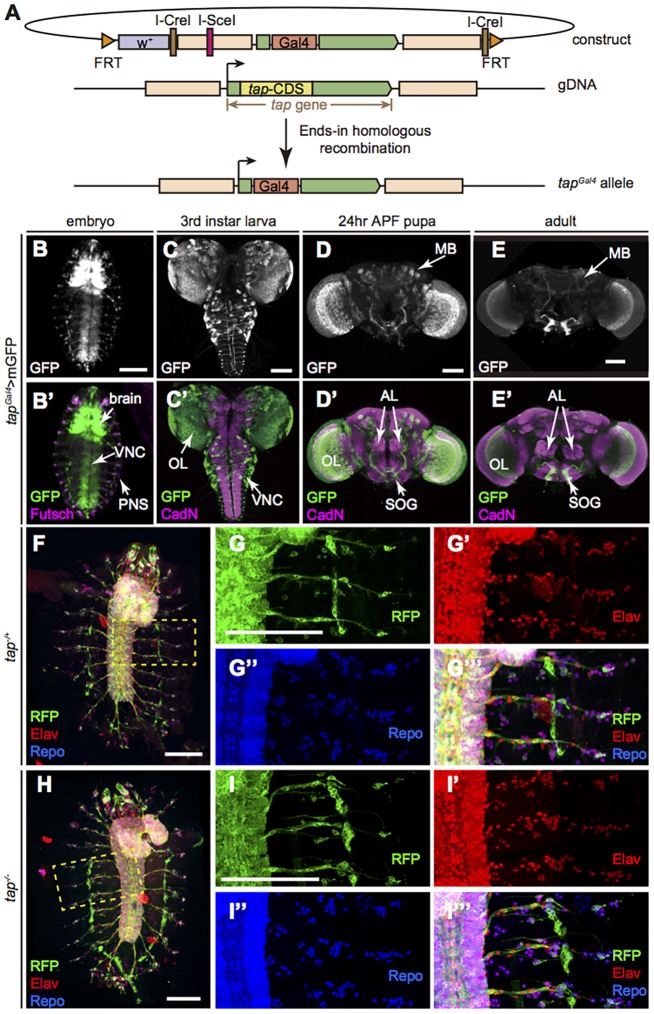
**Characterization of Tap expression patterns.** (A) Scheme for generation of the *tap^Gal4^* allele. (B-E′) Tap is widely expressed in the nervous system throughout development. Tap^+^ cells were labeled by membrane GFP (mGFP) driven by *tap^Gal4^*. (B,B′) Ventral view of a stage 16 embryo. (C,C′) L3 brain. (D,D′) Brain 24 h after puparium formation (APF). (E,E′) Adult brain. (F-I‴) Flat preparation of whole embryos at stage 17. Tap^+^ cells are labeled with CD8::RFP. The differentiated nervous system is visualized as neurons (red) and glia cells (blue). (F) *tap* heterozygous (*tap^Gal4^/+*) embryo. (G-G‴) Magnification of the boxed region in F. (H) *tap* null (*tap^Gal4/Gal4^*) embryo. (I-I‴) Magnification of the boxed region in H. AL, antenna lobe; MB, mushroom body; OL, optic lobe; PNS, peripheral nervous system; SOG, subesophageal ganglion; VNC, ventral nerve cord. Scale bars: 100 μm.

To investigate the function of Tap, we generated a mutant allele using ends-in homologous recombination (Rong and Golic, 2000) to replace the open reading frame of *tap* with an external driver, *Gal4* (Fig. 2A). The *tap^Gal4^* allele, in homozygosity, serves as a *tap* null mutant, while in heterozygosity it was used as a driver to reveal the expression pattern of Tap. We made several attempts to generate a Tap antibody, but this was not successful. Tap^Gal4^ was detected in a large number of cells in both the CNS and PNS throughout development (Fig. 2B-E). During embryogenesis, Tap is enriched in the ventral nerve cord and sparsely expressed in the PNS (Fig. 2B, Fig. S2), which is similar to the RNA distribution as revealed by *in situ* hybridization (Fig. S2). Postembryonically, Tap-positive cells were observed in many tissues, including optic lobe, mushroom body (MB), antenna lobe and subesophageal ganglion (Fig. 2C-E).

### Tap is not required for specifying the neuronal or glial cell fate during embryogenesis

Flies lacking Tap are mostly embryonic lethal, with a few escapers to early larval stages. This lethality can be rescued, including to adult viability, by re-expression of Tap (*UAS::tap*) in *tap^Gal4^* homozygous flies. We examined whether the number or fate of neuronal and/or glial cells are altered in *tap* mutant embryos. Surprisingly, we find no obvious morphological defects in the *tap* mutant embryos. The number and pattern of the neurons and glia in the PNS are intact. Although the cell number in the CNS is difficult to quantify, the general cellular pattern looked similar in mutant and control flies (Fig. 2F-I). These data suggest that Tap is not required for early neurogenesis in *Drosophila* embryos.

### *tap* mutants show MB β lobe midline crossing and an α lobe missing defect

**Fig. 3.**
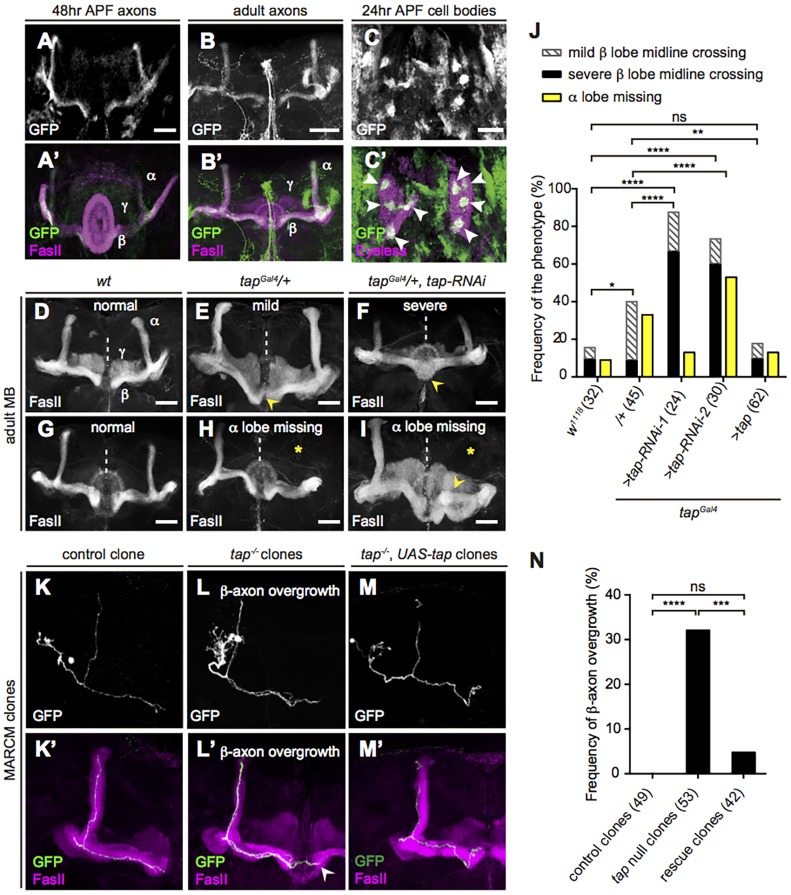
**Tap is required for the axonal growth and guidance of MB α/β neurons.** (A-B′) *z*-projection of MB axon level in 48 h APF pupa (A) or adult (B). FasII (magenta) strongly labels the α and β lobes and weakly labels γ lobes (A′,B′). GFP expression is restricted to a subset of axons in the distal region of α lobes and ventral region of β lobes. (C,C′) GFP is concentrated in four clusters of cells (arrowheads) within MB cell bodies (magenta). (D) Normal MBs in a wild-type fly. (E) A *tap* heterozygous mutant brain exhibits a mild β axon midline crossing defect in which the thickness of the fiber bundle crossing the midline is less than the width of the β lobe termini (arrowhead). (F) A *tap* RNAi knockdown MB exhibits a severe β lobe fusion defect in which the crossing fibers are equal in width to the terminal of adjacent β lobes (arrowhead). (G) Normal MBs in a wild-type fly. (H,I) *tap* mutants lose the dorsal projection of α/β neurons (asterisks). The loss of dorsal lobe is sometimes accompanied by the bifurcation of the medial lobe (arrowhead in I). (J) Fraction of brains that display defects. Number of brains analyzed is indicated in parentheses. ns, *P*=0.9242; *P=0.0280, **P=0.0083, ****P<0.0001; Chi-square test, two-tailed. (K-M′) One half of adult MBs. Axonal projections of labeled MB neurons generated by MARCM were visualized by GFP driven by *ey^OK107^-Gal4*. (K,K′) A control clone. (L,L′) *tap* null clones. β axons exceed the region of the β lobe, extending to the contralateral β lobe region (arrowhead). (M,M′) A rescue clone in which Tap is reintroduced into the mutant clonal cell only. (N) Fraction of clones that display β-axon overgrowth. Number of clones analyzed is indicated. ns, *P*=0.1224; ****P<0.0001, ***P=0.0009; Chi-square test, two-tailed. Scale bars: 50 μm.

To characterize Tap function, we focused on the MB as a model system. Tap is expressed in the MB at both pupal and adult stages in a subset of α/β neurons (Fig. 3A-C) that form four clusters (Fig. 3C) and project their medial axons to the dorsal part of the β lobe (Fig. 3A,B). Tap expression is highest at early pupal stages and then declines. The phase of high level Tap expression correlates with the differentiation stage of α/β neurons, indicating that Tap might regulate the development of the α/β lobe.

To circumvent embryonic lethality we began by exploiting the heterozygous *tap^Gal4^* allele alone or in combination with two independent RNAi strains targeting distinct regions of *tap*. In wild-type *Drosophila*, axons of the medially projecting β lobes terminate near the midline but do not cross it (Strausfeld et al., 2003). In contrast to the wild-type MB morphology (Fig. 3D), *tap* loss-of-function brains exhibit β lobe fibers that extend across the midline (Fig. 3E), sometimes causing fusion of the two contralateral β lobes (Fig. 3F). The phenotype is variable in severity; we classified it as ‘normal’, ‘mild’ or ‘severe’ based on the thickness and density of the β lobe fibers crossing the midline. *tap* heterozygotes display an increase in the penetrance of mild defects, while both *tap* RNAi strains raise the incidence and severity of the β lobe midline crossing defect. When Tap is re-expressed, the defect can be rescued to control levels (Fig. 3J). This β axon midline crossing defect is developmental in origin as it can be observed at early pupal stages (Fig. S3B). Moreover, ectopic expression of Tap throughout the entire MB or in all the Tap^+^ neurons increases the distance between two contralateral β lobes, suggesting that Tap induces the retraction of β lobes and/or inhibits the axonal growth of β lobes (Fig. S3D,E).

In addition to the β axon midline crossing defect, an ‘α lobe missing’ phenotype was observed in Tap loss-of-function MBs (Fig. 3H,I). In some brains with a missing α lobe, the β lobe appears to branch into two bundles (Fig. 3I), suggesting that this α lobe defect might be an axonal targeting defect rather than a growth defect. Unlike the β lobe defect, the penetrance of the α lobe missing phenotype varied considerably (Fig. 3J). Nonetheless, considering that the frequency of missing α lobes is still higher in the *tap* mutant flies than in wild-type and rescue strains, we conclude that Tap is essential for the growth and correct targeting of both lobes of α/β neurons.

### Tap is required cell-autonomously for the growth and targeting of the β lobe

Since the branching and growth pattern of single α/β axons cannot be directly inferred from the morphology of the α/β lobes, a single-neuron level analysis, such as mosaic analysis with a repressible cell marker (MARCM), is necessary to clarify the targeting of α/β neurons (Lee et al., 2000). Therefore, *tap* null clones were generated in a *tap^Gal4^* heterozygous background using the MARCM technique. In both wild-type control and *tap^Gal4^* heterozygous backgrounds, a small minority of brains showed β lobe overgrowth and/or the α lobe missing defect as discussed above. Considering this, only brains with intact overall α/β lobe morphology were quantified for clonal axon phenotypes. Analysis of small MB clones revealed that none of the control clones showed any defects (Fig. 3K). By contrast, in 32% of *tap* null mutant clones, β axons project beyond the β lobe domains (Fig. 3L,N). In severe cases, axons were observed to cross the midline and project to the contralateral β lobes. This defect can be rescued by re-introduction of *tap* specifically in the mutant clones (Fig. 3M,N). This suggests that Tap is required cell-autonomously for the development of the β axon branch. However, none of the mutant clones showed loss of α axon growth, suggesting that Tap plays a non-cell-autonomous role during α lobe development.

### Tap regulates axonal growth and guidance through Dsh

**Fig. 4.**
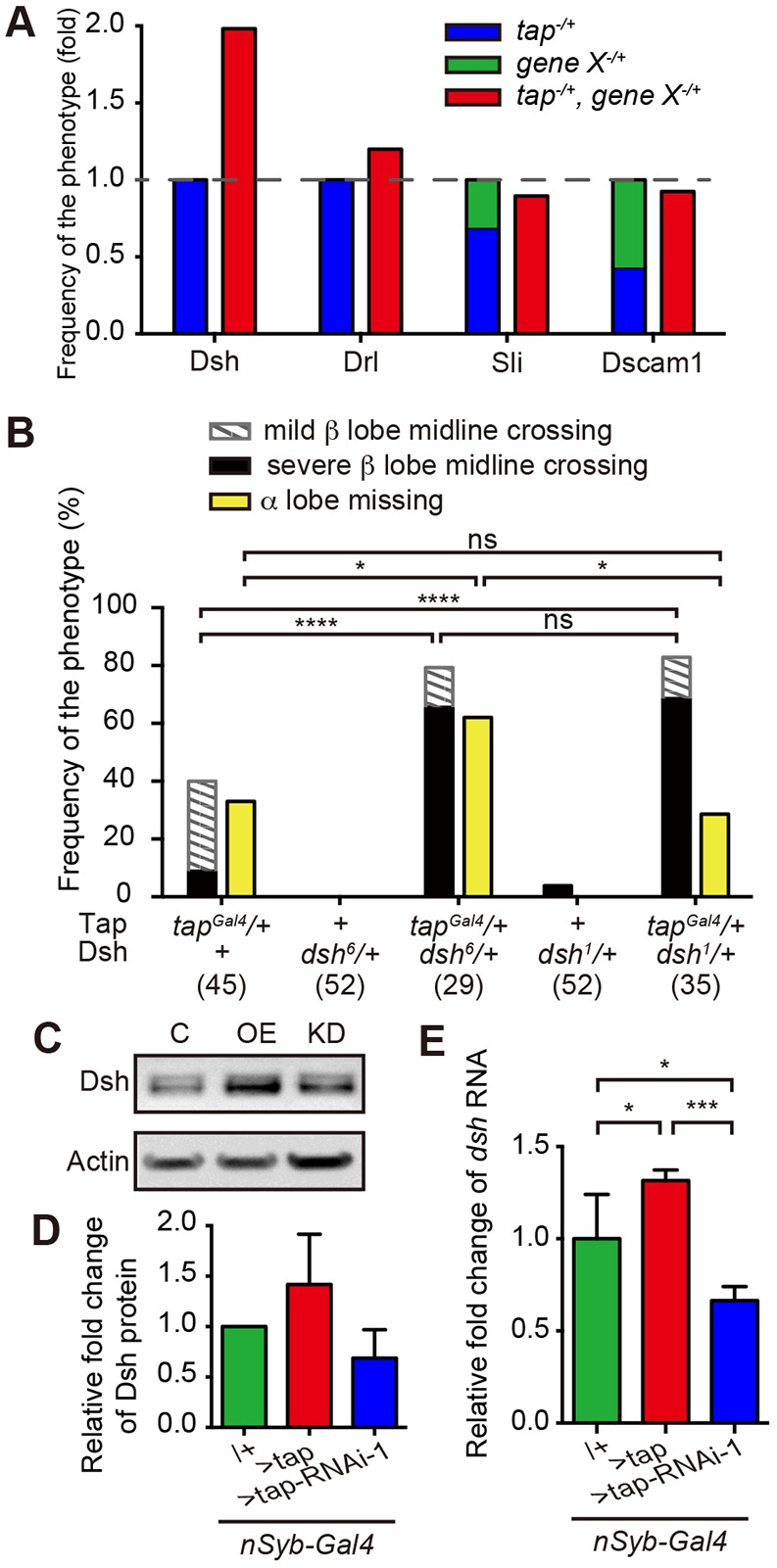
**Tap regulates axon guidance through Dsh in the PCP pathway.** (A) Genetic interactions for the β lobe overgrowth defect in double heterozygous mutants between *tap* and axon guidance factors known to cause β lobe overgrowth. The penetrance of the total β lobe overgrowth defect was normalized to the sum of the penetrance of the *tap* mutant plus that of the candidate gene mutant. Dashed line indicates the normalized defect severity aggregated by the two single mutants, set to 1. Number of brains analyzed: *tap^Gal4^/+*, 45; *dsh^6^/+*, 52; *tap^Gal4^/+, dsh^6^/+*, 29; *drl^Red2^*/+, 26; *tap^Gal4^/+,* *drl^Red2^*/+, 31; *sli^2^*/+, 23; *tap^Gal4^/+,* *sli^2^/+*, 27; *Dscam1^21^/+*, 26; *tap^Gal4^/+, Dscam1^21^/+*, 21. (B) Fraction of brains that display defects in *tap* and *dsh* double heterozygous mutants. Number of brains analyzed is indicated. *dsh^1^* is a PCP pathway-specific mutant; *dsh^6^* is a loss-of-function mutant. ns, *P*>0.5; *P<0.02, ****P<0.0001; Chi-squared test. (C) Western blot analysis showing Dsh levels in driver alone (lane C), or upon Tap overexpression (OE) or knockdown (KD). Actin is a loading control. (D) Quantification of relative changes in Dsh levels of OE or KD, normalized to control. Error bars indicate s.d., *n*=3. (E) qRT-PCR analysis showing *dsh* RNA level in driver alone, Tap OE or KD cases. Error bars indicate s.d., *n*=4; *P<0.05, ***P<0.001; one-way ANOVA, Tukey correction.

In order to investigate the molecular mechanism by which Tap regulates axonal growth and guidance, we performed dominant interaction tests between Tap and well-established MB axonal guidance factors, particularly those whose loss of function causes β lobe overextension. Specifically, we asked whether heterozygosity for any of these genes strongly enhances the very mild phenotypes observed in *tap* heterozygotes. Specifically, we looked for phenotypes significantly greater than the sum of the two phenotypes. Among the candidate genes, *drl* (Moreau-Fauvarque et al., 1998), *sli* (our unpublished data) and *Dscam1* (Hattori et al., 2007) did not show any obvious synergism with *tap* (Fig. 4A). However, loss of one copy of *dsh* strongly enhances both the α lobe guidance and β lobe overgrowth defects in the *tap* heterozygous background (Fig. 4A,B). This suggests that Dsh synergizes with Tap to regulate axon guidance and growth during MB development.

To test if Tap might regulate Dsh expression *in vivo* we overexpressed Tap or knocked it down in all neurons and measured Dsh protein and RNA levels. We find that Tap gain of function mildly increased Dsh levels, whereas Tap knockdown mildly decreased Dsh levels *in vivo* (Fig. 4C-E).

Dsh is a key Wnt signaling component and serves as a hub to relay signals from the receptors to the downstream effectors (Gao and Chen, 2010). Dsh is essential for both canonical and non-canonical Wnt pathways, such as the Wnt-PCP pathway. In *Drosophila*, components of the PCP pathway are known to regulate distinct phenomena, including polarity within cell sheets (Fanto and McNeill, 2004), dendritic arborization (Gao et al., 2000) and axonal growth and guidance (Ng, 2012); and the role of Dsh in MB β lobe axonal growth is mediated by its activity in the Wnt-PCP pathway (Shimizu et al., 2011). To further query the interaction between Tap and Dsh we took advantage of the Wnt-PCP-specific mutant *dsh^1^*. Interestingly, the combination of *dsh^1^* and *tap* heterozygosity increases the severity of the β lobe overgrowth defect to a level similar to that of *tap* and *dsh^6^* (a null allele) heterozygosity, but does not affect the α lobe missing defect (Fig. 4B). These data suggest that the role of Tap in the regulation of axonal growth and guidance in the α lobe and the β lobe are independent. Furthermore, this is consistent with previous findings that Wnt-PCP signaling regulates β lobe growth cell-autonomously, but α lobe growth non-cell-autonomously (Soldano et al., 2013). Unexpectedly, ectopic expression of Dsh also increases the β lobe overgrowth defect (Fig. S4A). Furthermore, overexpression of a Wnt-PCP mutant form of Dsh results in a similar severity of defects to overexpression of wild-type Dsh (Fig. S4A).

In addition to Dsh, other key Wnt-PCP components are known to regulate α/β lobe growth and guidance, namely Abl, Appl, Stan, Vang and Wnt5 (Shimizu et al., 2011). Heterozygosity for these genes had a very mild effect on the *tap* defect and none of them changed significantly in terms of expression level upon manipulation of Tap expression (Fig. S4B,C).

Our data are consistent with the idea that Tap functionally interacts with the Wnt-PCP pathway to regulate neuronal extension and guidance. Cellular polarity created by the PCP pathway is essential to direct cell movement, which is analogous to the movement of growth cones (Zou, 2012). Most of the known regulation of the Wnt-PCP pathway is via protein-protein interactions. For instance, Vangl2, a core receptor of the PCP pathway in mammalian cells, was found to antagonize Dvl, the mammalian homolog of *Drosophila* Dsh, and to post-translationally regulate another receptor protein, frizzled 3, to regulate the polarity of growth cones in the filopodia (Shafer et al., 2011).

Here we identify a transcriptional modulator of the PCP pathway during axonal growth. Both mRNA and protein expression of *dsh* are modified upon Tap manipulation, although the modification is mild, suggesting that Tap serves as a regulator rather than an activator of *dsh*. Our genetic data also point to a possible complex feedback regulation, as both increased and decreased Dsh levels seem to enhance Tap loss-of-function phenotypes. It is curious that Tap plays a non-cell-autonomous role in MB α lobe growth. This suggests that the development of the α and β lobes are regulated independently. The fact that the null allele of *dsh*, but not the ‘PCP-specific’ allele, causes this effect could suggest the involvement of the canonical Wnt pathway in α lobe growth.

In contrast to the potential conservation of its function in neurite growth, Tap does not act as a proneural protein in flies. Despite the obvious differences in morphology between invertebrates and vertebrates, the fundamental mechanisms underlying neurogenesis are conserved. In particular, the induction of neurogenesis by bHLH proteins has been conceptually defined as a module that can be applied to various contexts (Schlosser and Wagner, 2004). Our data, together with previous studies, suggest that during the evolution of vertebrates and invertebrates from their last common ancestor, the ancestral vertebrate neuroectoderm, but not that of invertebrates, became responsive to the neuroinductive activity of neurogenin-like proteins. It will be very interesting to determine whether the activity of ancestral-like proneural proteins, such as those found in sponges, resembles that of neurogenin, atonal or achaete-scute proteins. Crucially, this is not encoded as a change in the inductive capacity of the invertebrate neurogenins per se, as demonstrated by the neurogenic activity of Tap in *Xenopus*. Whether this is related to the dorsoventral axis switch in the location of the neuroectoderm (dorsal in vertebrates, ventral in *Drosophila*) remains to be determined.

## MATERIALS AND METHODS

### Fly husbandry and transgenic lines

Flies were kept at 25°C or 29°C on standard medium. Experiments involving RNAi or genetic interaction were performed at 29°C, and remainder at 25°C. Transgenic lines are listed in the supplementary Materials and Methods.

### *Xenopus* embryo microinjection

*ato*, *tap* and mouse *Ngn1* mRNAs were injected into a single blastomere of *Xenopus* embryos at the two-cell stage. Whole embryos were *in situ* hybridized with an *N-tubulin* probe as described (Quan et al., 2004).

### Cloning and gene targeting

Fragments comprising the 5′ homologous recombination arm of the *tap* ORF, *Gal4* and the 3′ recombination arm of the *tap* ORF were amplified using the primers listed in the supplementary Materials and Methods and subcloned into pED13(M) vector (a gift of the B. Dickson laboratory, Janelia Farm Research Campus). *tap* targeting was achieved by ends-in homologous recombination (Fig. S1) (Rong and Golic, 2000).

### Immunohistochemistry

Embryos and dissected tissues were stained using a published protocol (Hassan et al., 2000; Langen et al., 2013). Flat preparations of embryonic fillets at stage 17 were generated and stained on poly-lysine-coated glass following the protocol described by Featherstone et al. (2009). Images were acquired using a Leica TCS SP8 or SP5 and processed with ImageJ (NIH). Antibodies are detailed in the supplementary Materials and Methods.

### *In situ* hybridization

Embryos and L3 brains were hybridized as described (Hassan and Vaessin, 1997). *tap* cDNA was used to generate the digoxigenin-labeled antisense probe.

### Western blot

Proteins from 20 adult fly brains of each genotype were resolved and probed using the protocol of Okray et al. (2015). Antibodies are detailed in the supplementary Materials and Methods.

### qRT-PCR

qPCR was performed according to the protocol of Li et al. (2013). A hundred adult fly heads were collected for each genotype. *Rp49* (*RpL32*) and *RpS13* were used as reference genes, and qbase+ (Biogazelle) was used to process the data.
